# Refusal of Therapy: When Is It Appropriate?

**DOI:** 10.6004/jadpro.2015.6.2.1

**Published:** 2015-03-01

**Authors:** Pamela Hallquist Viale

In our last issue, I talked about care in the context of end of life, in particular the expense and futility of care given in the last months of life or when patients are facing an incurable condition. I believe that it is entirely appropriate to refuse care or decline aggressive therapies at the end of life.

Recently, the news has been full of reports detailing the story of Cassandra Fortin, a 17-year-old girl from Connecticut diagnosed with Hodgkin lymphoma this past September. Cassandra (with her mother’s support) refused therapy for her potentially curable cancer. The Connecticut Supreme Court recently ruled unanimously to uphold the state’s decision to place the teenager in the custody of child welfare authorities (Larimer, 2015). She was forced to undergo placement of an implanted port and receive chemotherapy that has approximately an 85% chance of saving her life (Nalpathanchil, 2015). Cassandra’s reasons for wanting to refuse therapy? She feels that chemotherapy is poison, and she doesn’t want the toxins in her body. Her mother supports the teenager’s position and feelings, and the two want to explore alternative therapies for her lymphoma.

## MEASURING MATURITY

Teenagers are known for many things, but maturity is not generally one’s first thought when referring to this age group. Teenagers often exhibit risk-taking behavior, although we’ve yet to discover a concrete biological explanation for these actions. Historical precedence shows differences in societal measurement of maturity (Johnson, Blum, & Giedd, 2009). For example, 21 is now the legal drinking age in the United States. Individuals must be of a certain age to hold specific US offices. Here, a person must be 35 to be president, yet in the United Kingdom one can stand for election at 18 (Johnson, Blum, & Giedd, 2009). Crimes committed by adolescents often receive less harsh judgments, in part because the age of the individuals affects their perceived responsibility for their actions. Society, in general, expects that individuals who are engaged in certain activities should have the maturity to handle the decisions and actions inherent in those activities.

There are many aspects unique to the adolescent brain. Studies have demonstrated that the adolescent brain continues to evolve and change past the teens (Johnson, Blum, & Giedd, 2009). Scans of teens have demonstrated that the volume of gray matter changes, occurring through age 20. Gray matter forms the cortex of the brain and is the repository of thought and memory processes. Studies have shown that the parts of the brain responsible for impulse behavior and the ability to plan ahead (typical adult behaviors) mature toward the end of the brain’s development (National Institute of Mental Health [NIMH], 2011). The brain is also affected by hormonal changes in adolescence that can have significant effects on behavior (NIMH, 2011).

In my youth, my parents saw me make a number of poorly thought-out decisions, but most of those mistakes could be easily rectified (a different job, a change in school classes, a new boyfriend). However, the decision Cassandra faces is exponentially more serious. If she doesn’t receive the appropriate therapy, Cassandra will most likely die of a potentially curable disease.

## DELAYING TREATMENT IN FAVOR OF ALTERNATIVE THERAPIES

Alternative therapies can seem very attractive to patients. One of the most intriguing aspects of these therapies is the idea that alternative treatments are "less toxic" than traditional therapies. I certainly agree that traditional chemotherapy can be toxic and often causes significant side effects for patients. But I’m a believer in evidence-based medicine. I think tumor treatment should be based on the evidence gained from multiple clinical trials designed to demonstrate effectiveness of therapies for different cancers. If an alternative therapy has substantial evidence in treatment of a specific tumor type, then I would be in favor of adding it to the standard of care. If there is no substantial evidence but anecdotal evidence of helpfulness, I would consider adding it to the standard of care *if no harm would occur* as a result of the change. But replacing the evidence-based standard of care with alternative therapy alone without evidence? That is not a decision that I could personally choose or support.

If an adult patient chose to make the above decision rather than undergoing traditional, evidence-based care, I would educate, counsel, and cajole, but I would ultimately respect the adult patient’s choice. However, when an adolescent makes that choice, I have to side with the Connecticut Supreme Court: Cassandra lacks the maturity to make such a decision.

## CHOICES REGRETTED

Steve Jobs, the co-founder of Apple, died of a rare pancreatic cancer in 2011. His biographer told the media that Jobs regretted his decision to refuse surgery and undergo alternative herbal treatments for his disease, especially when his health declined (Potter, 2011). Jobs had control over what happened to his body, and his death, although tragic, may have been partially due to his decisions regarding the delay in receiving standard therapy. But as an adult, Jobs had the power to choose. In Cassandra’s case, the court is looking after her best interest, giving her the best chance to become an adult and live a cancer-free life. I agree with its decision.

## EARN CE IN EACH ISSUE OF JADPRO!

I’ve always been proud of the level of education found in articles we’ve published in JADPRO. But now I’m excited to announce that going forward, at least one article in each issue of JADPRO will be associated with free CE/CME/pharmacy credits! Read the Grand Rounds article by Malangone et al., then follow the instructions on how to earn your free credit and receive your certificate.
